# Mediterranean diet research trajectories in China (2006–2025): a scoping review and scientometric analysis to localize global nutrition models

**DOI:** 10.3389/fnut.2025.1661835

**Published:** 2025-09-24

**Authors:** Dongming Jia, Shengxia Xue

**Affiliations:** ^1^Zhejiang Police Vocational College, Hangzhou, Zhejiang, China; ^2^The Third People's Hospital of Yuhang District, Hangzhou, Zhejiang, China

**Keywords:** Mediterranean diet, bibliometric analysis, Jiangnan diet, sustainability, digital health, China

## Abstract

**Background:**

Mediterranean-diet (MedDiet) principles are increasingly invoked to counter China’s nutrition-transition-driven epidemic of cardiometabolic disease, yet no field-wide synthesis of this scholarship exists. A bibliometric assessment can expose thematic evolution, knowledge gaps and localization pathways.

**Methods:**

We systematically searched Web of Science Core Collection and CNKI for Chinese-affiliated MedDiet human-health articles published between 2006 and 2 February 2025. After PRISMA-ScR screening we retained 384 records. VOSviewer and COOC mapped co-authorship, citations and keywords; latent-Dirichlet allocation detected topic drift; compound annual growth rate (CAGR) and field-weighted citation impact (FWCI) indexed performance. All data and scripts are openly archived.

**Results:**

Annual output climbed from one article in 2006 to 76 in 2022 (CAGR = 23%); FWCI = 1.34. Seven keyword clusters now pivot on gut-microbiome science, digital adherence and sustainability rather than early cardiometabolic replication. Temporal segmentation revealed three phases: replication (2006–2013), public-health expansion (2014–2019) and cross-disciplinary innovation (2020–2025). Bibliographic coupling resolved five citation schools; Jiangnan-diet localization has migrated into the leading clinical cluster. Lexical drift highlights ingredient substitution (rapeseed-oil phenolics) and late adoption of carbon-footprint terminology.

**Conclusion:**

Chinese MedDiet scholarship is recalibrating toward a culturally adapted, digitally enabled and climate-aligned paradigm. Longer m-health-supported trials, life-course epidemiology and multi-omics Jiangnan cohorts are warranted to translate current bibliometric momentum into population-level health and sustainability gains.

## Background

The Mediterranean diet (MedDiet), first systematically described by Ancel Keys in the 1950s, has long been associated with reduced mortality from coronary heart disease and other non-communicable diseases (NCDs) (1). Characterized by high consumption of plant-based foods, olive oil as the primary fat source, moderate intake of fish and poultry, and minimal red meat, the MedDiet has been consistently linked to lower risks of cardiovascular disease, type 2 diabetes, and certain cancers (2). Subsequent developments, including the 2010 MedDiet pyramid and the MedDiet 4.0 framework, have expanded its relevance to include cultural heritage, frugality, and environmental sustainability, positioning it as a globally endorsed model for health-promoting and climate-aligned nutrition (3).

The Mediterranean diet is defined not only by its distinctive food pattern but also by its nutrient and bioactive composition. High consumption of fruits, vegetables, and legumes provides abundant antioxidants, including polyphenols, carotenoids, and vitamins C and E, which mitigate oxidative stress and systemic inflammation (4, 5). Omega-3 fatty acids from fish and nuts are pivotal for cardiometabolic and cognitive health (6), while monounsaturated fats from olive oil contribute to favorable lipid profiles (7). Whole grains and legumes further supply dietary fiber that supports glycemic control and modulates the gut microbiota (8). These nutritional underpinnings explain the Mediterranean diet’s broad clinical and public health relevance and provide a scientific rationale for its cultural adaptation in non-Mediterranean settings.

China, by contrast, is undergoing a rapid nutrition transition. Since the 1990s, the dietary landscape has shifted markedly toward energy-dense, animal-based and ultra-processed foods. As of 2020, over half of Chinese adults were living with overweight or obesity, accompanied by rising prevalence of diabetes and cardiovascular conditions (9, 10). Popkin’s dietary modernization framework aptly characterizes this evolution toward obesogenic food environments. These epidemiological trends have prompted Chinese researchers and policymakers to explore whether MedDiet principles can be effectively transplanted—and culturally adapted—within East Asian dietary patterns.

Growing scholarly interest supports this hypothesis. A bilingual literature search of Web of Science and CNKI identified 384 MedDiet-related human-health articles published in China between 2006 and early 2025, with annual output rising from two publications in 2006 to a peak of 75 in 2022. A promising avenue for localization is the Jiangnan Diet, which emphasizes rice, vegetables, freshwater fish, and tea—culinary features that resonate with MedDiet principles while reflecting regional traditions (11–13).

Despite this progress, existing literature remains fragmented and uneven in thematic scope. Most studies focus on short-term clinical endpoints or nutrient adaptation, while several critical gaps persist: the absence of longitudinal and field-wide mapping, insufficient theorization of cultural translation, limited use of digital tools for adherence promotion, and minimal assessment of environmental co-benefits in China’s policy discourse (14).

Guided by acculturation theory—which conceptualizes foreign health practices as culturally reinterpreted frameworks—and by lifestyle-transition theory, which links behavior change to socioeconomic transformations (15), we propose that the MedDiet in China should be viewed not only as a nutritional intervention but also as a sociocultural phenomenon in transition.

This study employs bibliometric and semantic network analyses to map the evolving structure of Chinese MedDiet scholarship (2006–2025), addressing the following research questions:

**RQ 1**: How have the volume, thematic priorities, and intellectual structure of Chinese MedDiet research evolved over time?
**RQ 2**: Which institutions, author networks, and regional hubs shape this scholarship, and how do they frame the MedDiet’s cultural adaptation and policy relevance?

In answering these questions, we aim to provide the first comprehensive mapping of how a globally established dietary paradigm is being reimagined to address China’s dual imperatives of public health and environmental sustainability.

## Methods

### Study design and protocol

This investigation is a systematic scoping review and bibliometric analysis conducted in accordance with the PRISMA Extension for Scoping Reviews (PRISMA-ScR) (16).

### Eligibility criteria (PICOS)

**Population**: Publications with at least one mainland-China institutional address that investigate the Mediterranean diet.

**Intervention**: Conceptualization or application of Mediterranean-diet principles, including Jiangnan-diet localization.

**Comparators**: Not applicable; field-level scoping review without comparative arms.

**Outcomes**: Bibliometric indicators (publication volume, citation impact, thematic clusters) and reported health domains (cardiometabolic, microbiome, sustainability).

**Study designs**: Original research articles, reviews, guidelines and clinical trials indexed in Web of Science Core Collection (WoS) and China National Knowledge Infrastructure Core Collection (CNKI) between 1 January 2006 and 2 February 2025 (17). Editorials, letters, basic laboratory studies without human subjects, animal experiments and single-case reports were excluded.

### Information sources and search strategy

A bilingual search query was used for each database to maximize inclusivity. For Web of Science, the following syntax was applied: (TS = (Mediterranean diet) AND CU=CHINA) AND (DT==(“ARTICLE” OR “REVIEW”)), Timespan: 2006-01-01 to 2025-02-02 (Publication Date) | Exact search. For CNKI, the equivalent Chinese query was zhuti = “Dizhonghaiyingshi.” The search results were exported in UTF-8 encoding as .txt files (WoS) and .nbk files (CNKI).

### Selection process and eligibility criteria

Duplicate removal was conducted using Bibliometrix (R, version 4.2), which employs an automated author–title matching algorithm to identify duplicate records across databases. All flagged duplicates were manually verified by two independent reviewers to ensure accuracy.

Eligibility screening was performed in two stages, following PRISMA-2020 guidelines (18). In the first stage, database filters were used to exclude records that were not original research articles, such as reviews, editorials, letters, or commentaries. In the second stage, two independent reviewers assessed the titles and abstracts (and full texts where necessary) according to a predefined PEO framework: Population = humans in China; Exposure = Mediterranean diet; Outcomes = health, sustainability, or adherence-related measures.

Exclusion criteria were: (i) editorial content or commentaries; (ii) basic laboratory studies without human subjects; (iii) animal experiments; and (iv) single-case reports. Any disagreements were resolved by consultation with a third reviewer.

### Data items and data charting process

We extracted the following variables from each included record: (i) Bibliographic metadata: author(s), year of publication, title, journal, country/region of affiliation, institutional address, and document type; (ii) Study characteristics: health domain (e.g., cardiometabolic, microbiome, sustainability), study design (original research, clinical trial, review, guideline), and population focus; and (iii) Keyword and thematic variables: author keywords, MeSH-standardized keywords, and thematic cluster assignment.

Data charting was performed using a standardized extraction form that was pre-tested on a 5% sample of records to ensure consistency. Two reviewers (JD and SX) independently charted all data in duplicate. Any discrepancies were resolved through discussion until consensus was reached. No additional contact with study authors was required to obtain or confirm data.

### Data synthesis

#### Data cleaning and Preprocessing

All records were imported into Bibliometrix (R version 4.2) for merging and standardization. Author disambiguation—such as distinguishing “Li Y.” from “Li, Yong”—was achieved using ORCID identifiers and institutional affiliations. Keyword harmonization was performed using the Medical Subject Headings (MeSH) thesaurus and the Chinese Nutrition Terminology List. For instance, “NAFLD” was standardized to its full term, “non-alcoholic fatty liver disease.”

#### Bibliometric and network analyses

A multi-layered bibliometric and semantic-network analysis was conducted to explore the intellectual structure and thematic evolution of the field. VOSviewer (version 1.6.20) was used to construct co-authorship networks (minimum threshold: three documents), co-citation clusters (threshold: 20 citations), and bibliographic coupling maps for identifying citation communities (18). In parallel, COOC software (version 14.3) generated term-frequency heatmaps, phase-specific co-occurrence matrices, and co-word networks to detect semantic drift over time (19).

Core bibliometric indicators included total link strength (TLS), betweenness centrality, field-weighted citation impact (FWCI), and compound annual growth rate (CAGR). Two additional techniques were employed to deepen the analysis. First, bibliographic coupling analysis (BCA) was applied by clustering articles that shared 20 or more references, revealing foundational intellectual subfields (20). Second, term co-occurrence analysis (TCA) was used to identify frequent keyword pairings (≥5 papers), thereby capturing shifts in topical focus over time (21).

#### Topic modeling

To model the latent thematic structure of the literature, we applied Latent Dirichlet Allocation (LDA) using Gensim (version 4.3.2). The modeling corpus consisted of English abstracts and Google-translated Chinese abstracts. The quality of translation was validated by manually reviewing 10% of the translated corpus, with a verified error rate below 2%. The number of topics was optimized via grid search over values of K from 5 to 15, using C_V coherence as the objective function, which peaked at K = 7 (C_V = 0.53). The final LDA model was run with *α* = 0.01, *β* = 0.10, 1,000 iterations, 10 passes, and a fixed random seed of 42 (22).

#### Reliability and sensitivity analyses

To ensure methodological robustness and reproducibility, three forms of validation were conducted. First, a subset replication analysis re-applied the full bibliometric and LDA workflows to (i) a WoS-only subset and (ii) an English-only subset. Resulting differences in cluster composition were less than 5%, and modularity score deviations were below 3%, indicating minimal sensitivity to language or database origin. Second, a split-sample validation was conducted by manually recoding a random 10% of the dataset, resulting in a keyword frequency deviation below 1.2% and thematic assignment consistency of *κ* = 0.89. Third, a reproducibility audit was implemented via a continuous integration (CI) pipeline. All R and Python scripts were version-controlled on GitHub, and automated tests for data import, cleaning, and visualization were executed through GitHub Actions at each commit.

### Risk-of-bias assessment

Formal risk-of-bias tools (e.g., RoB 2, AMSTAR-2) are designed for clinical outcomes and are inapplicable to bibliometric metadata. Instead, potential biases—language restriction, database coverage and affiliation misclassification—are detailed in the Limitations subsection.

### Ethics statement and data availability

This scoping review was conducted using publicly available data from previously published studies and did not involve human participants, identifiable personal data, or interventions. Therefore, approval from an ethics committee was not required. All procedures followed the principles of responsible research and publication ethics in accordance with the guidelines of the Committee on Publication Ethics (COPE).

The authors affirm that this work is original, free from plagiarism or duplicate publication, and that all sources have been properly acknowledged. All listed authors meet established authorship criteria. Any conflicts of interest have been fully disclosed. The authors also commit to correcting or retracting the work if any major errors or misconduct are identified. All datasets and analysis scripts are publicly archived and available.

## Results

### Evidence selection

A total of 970 records were identified through database searching (Web of Science = 866; CNKI = 104). After automated and manual duplicate removal (*n* = 19) and automated ineligibility screening (*n* = 17), as well as removal of unrelated or incomplete records (*n* = 418), 516 records were retained for screening. Of these, 59 were excluded based on title/abstract assessment, leaving 457 full-text articles for eligibility assessment.

**Figure 1:**
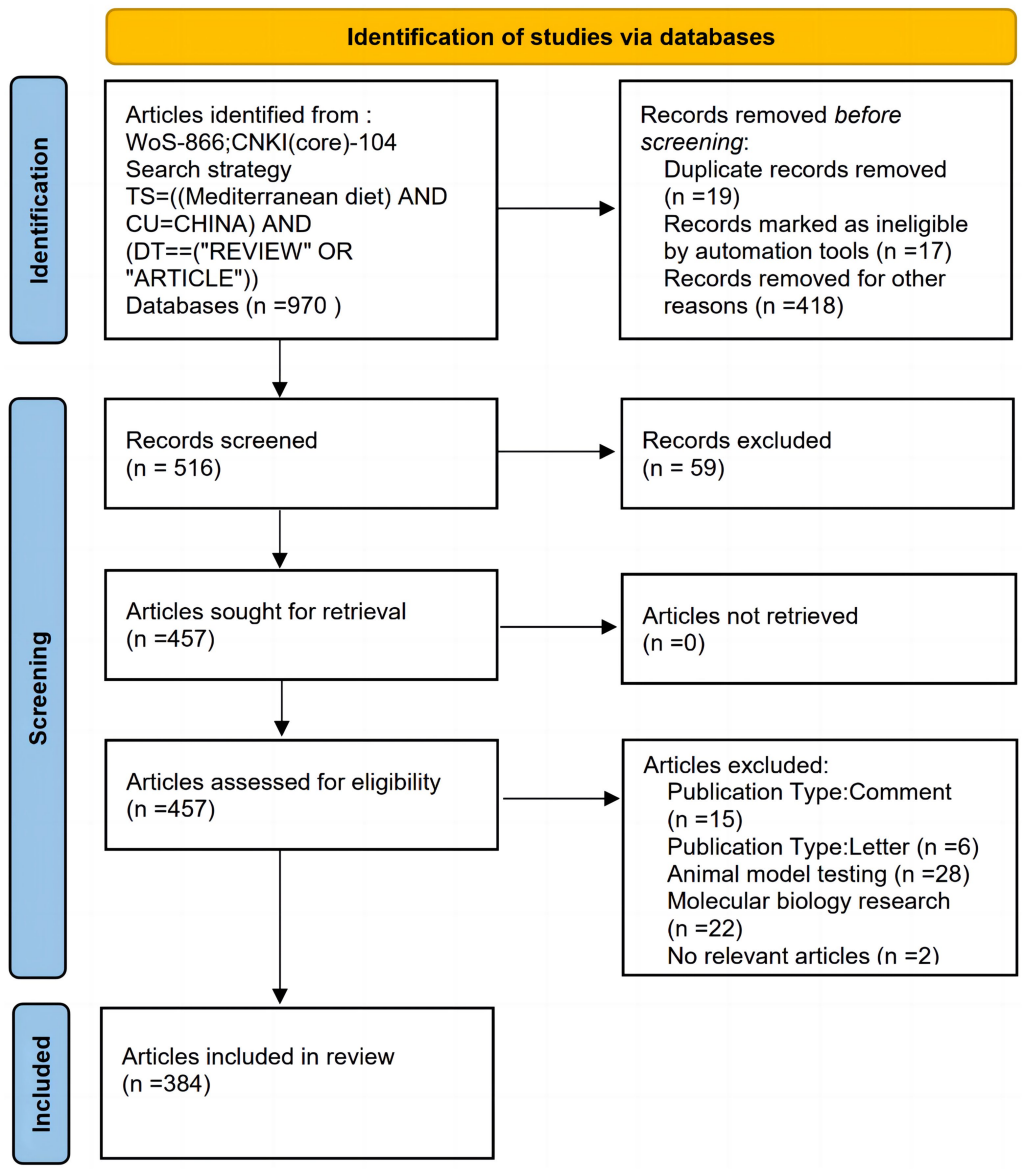
Flow chart depiction of the research methodology. (1) WoS, Web of Science; (2) CNKI, China National Knowledge Infrastructure.

During full-text eligibility screening, the following were excluded: Commentaries (*n* = 15); Letters (*n* = 6); Animal model testing (*n* = 28); Molecular biology research without human participants (*n* = 22); No relevant content (*n* = 2). This yielded a final set of 384 articles for inclusion. Inter-rater reliability for screening was high (Cohen’s *κ* = 0.87). The complete selection process is presented in Figure 1.

### Publication output and growth trajectory

**Figure 2:**
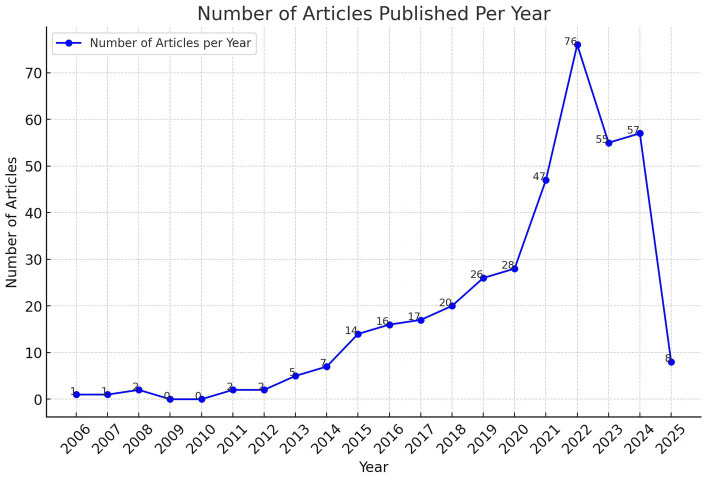
Annual publication trends of Mediterranean-diet research in China (2006–2025).

Between 2006 and the search cut-off on 2 February 2025, a total of 384 eligible articles on Mediterranean diet research in China were identified. The annual publication volume increased substantially, from just one article in 2006 to a peak of 76 in 2022, corresponding to a CAGR of 23%. Output declined modestly to 55 articles in 2023 and stabilized at 57 in 2024, suggesting a pandemic-related surge followed by a post-pandemic plateau in scholarly production (Figure 2).

Citation metrics followed a similar upward trend. By 2023, the cumulative citation count surpassed 4,000, and the FWCI for the corpus reached 1.34, indicating that Chinese MedDiet articles are cited 34% more frequently than the global medicine baseline (data available in Supplementary Table S1). These Figures underscore both the quantitative expansion and qualitative impact of MedDiet scholarship in China over the past two decades.

### Leading institutions, authors, and geographic hubs

Chinese Mediterranean diet research is primarily concentrated in coastal academic clusters. Table 1 highlights the three leading regional hubs—Beijing, Hong Kong, and the Zhejiang–Shanghai axis—which together account for 33% of the total publications and 38% of all citations. Peking University leads in output with 49 articles, while the Zhejiang–Shanghai collaboration (Zhejiang University and Fudan University) achieves the highest citation count (559) and the greatest TLS (412), indicating a central position in the national collaboration network.

**Table 1:** Productivity and influence of the three leading institutional hubs (2006–2025).

Hub	Core institution(s)	Documents	Citations	Total link strength*
Beijing	Peking U., Beijing Hosp., Capital Med. U.	49	312	388
Hong Kong	CUHK, HKU	43	298	274
Zhejiang–Shanghai	Zhejiang U., Fudan U.	35	559	412

^*^ Total-link strength (TLS) extracted from VOSviewer co-authorship network (threshold ≥3 documents).

At the author level, trans-Pacific collaborators rank highest in citation density. Willett W.C. (Cit/Doc = 73.2) and Pan A. (63.2) exemplify this trend through their extensive U.S.–China cohort studies. Network analysis identifies Li Y. (betweenness centrality = 0.19) and Woo J. (0.17) as strategic “boundary spanners” who connect the Beijing and Hong Kong clusters, respectively, enhancing national knowledge diffusion (Supplementary Table S1).

**Figure 3:**
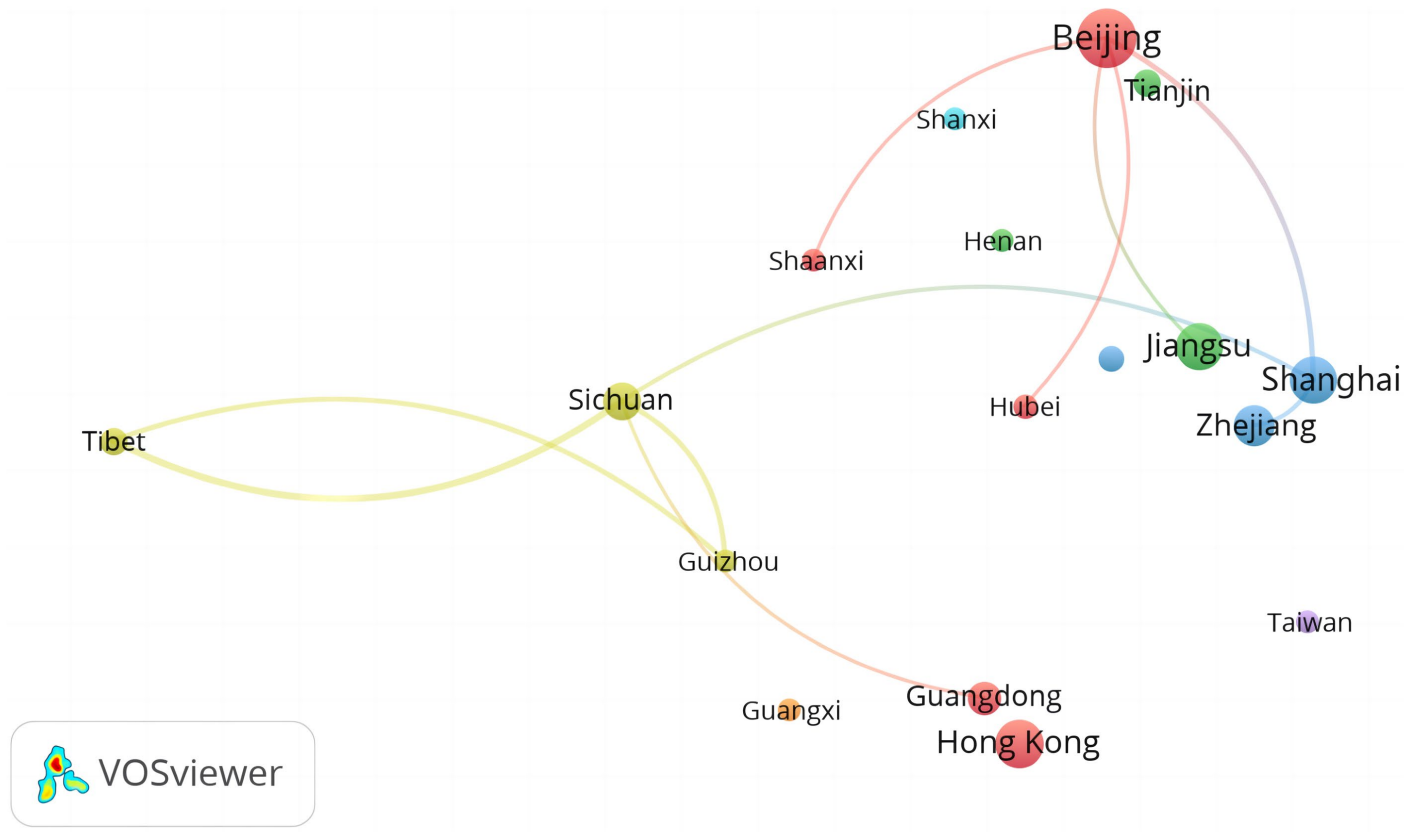
Inter-provincial co-authorship network of Mediterranean-diet research in China (2006–2025). Node size, number of publications from the province; edge width, co-authored papers between province pairs. Colors correspond to modularity classes produced by the VOSviewer Louvain algorithm.

Geographic collaboration patterns show an intensifying east–west knowledge flow. Figure 3 presents the inter-provincial co-authorship network, with node size proportional to publication volume and edge width indicating co-authorship frequency. Notably, the proportion of edges linking inland provinces increased from 31% during Phase I (2006–2013) to 44% during Phase III (2020–2025), suggesting a gradual diffusion of research capacity beyond coastal strongholds—likely reflecting policy initiatives such as Healthy China 2030 that incentivize regional research partnerships.

### Keyword structure: seven thematic clusters

**Figure 4:**
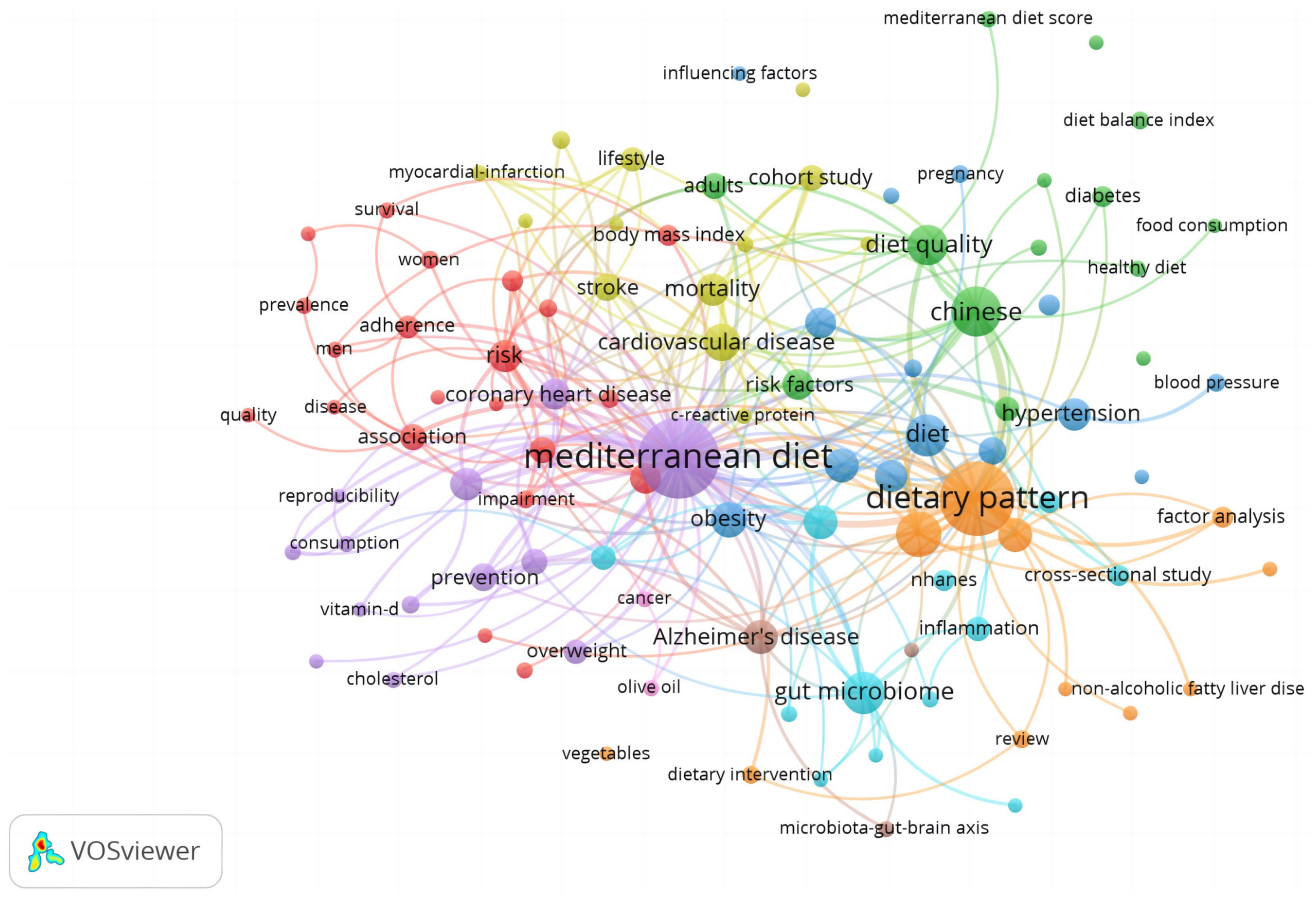
Keyword co-occurrence network of Mediterranean-diet research in China (2006–2025).

Author-keyword co-occurrence analysis (threshold:≥5 appearances) identified 80 high-frequency terms, which were grouped into seven coherent thematic clusters using association-strength normalization in VOSviewer. These clusters account for 81% of TLS in the corpus. Clustering quality was deemed acceptable (Louvain modularity = 0.46; average silhouette = 0.71). The network map is shown in Figure 4, and quantitative details appear in Table 2.

**Table 2:** Keyword clusters in Mediterranean-diet research in China (2006–2025).

Cluster ID	Color ^†^	One-line Theme	Top keywords ^‡^ (≥ 15 occurrences)	Terms in cluster (n)	Share of total link strength (%)
1	Purple	Mediterranean diet and overall preventive effects	Mediterranean diet; prevention; consumption	14	22
2	Red	Cardiovascular-risk factors and dietary adherence	Risk; coronary heart disease; adherence	16	18
3	Orange	Dietary pattern—gut microbiome / inflammation	Dietary pattern; gut microbiome; inflammation	12	14
4	Green	Chinese diet quality and diabetes	Chinese; diet quality; diabetes	11	13
5	Blue	Metabolic-syndrome-related dietary studies	Hypertension; diet; blood pressure	10	10
6	Yellow	Epidemiological design & risk factors	Cardiovascular disease; mortality; stroke	9	15
7	Teal	Gut–brain axis, obesity & interventions	Alzheimer’s disease; microbiota-gut-brain axis; dietary intervention	8	8

† Colors correspond to node colors in Figure 4. ‡ Only the three highest-frequency keywords per cluster are listed.

Cluster 1 (purple) centers on the term “Mediterranean diet” and encompasses prevention-oriented research addressing chronic disease risk reduction. Cluster 2 (red) focuses on cardiovascular endpoints (e.g., coronary heart disease, risk), often linked with adherence—indicating a shift from epidemiological replication to behavioral implementation.

Microbiome research forms two distinct clusters: Cluster 3 (orange) links dietary patterns to gut microbiota and inflammation pathways, while Cluster 7 (teal) extends to the gut–brain axis, obesity, and Alzheimer-related outcomes. Cluster 4 (green) captures localized adaptations, with terms such as “Chinese,” “diet quality,” and “diabetes,” reflecting attempts to recalibrate MedDiet metrics to native food patterns. Cluster 5 (blue) emphasizes hypertension and blood pressure, whereas Cluster 6 (yellow) features mortality-focused cohort studies. Together, these clusters demonstrate a thematic diversification from cardiometabolic endpoints to digital adherence, microbiomics, and contextual adaptation.

### Phase-wise topic evolution (2006–2025)

**Figure 5:**
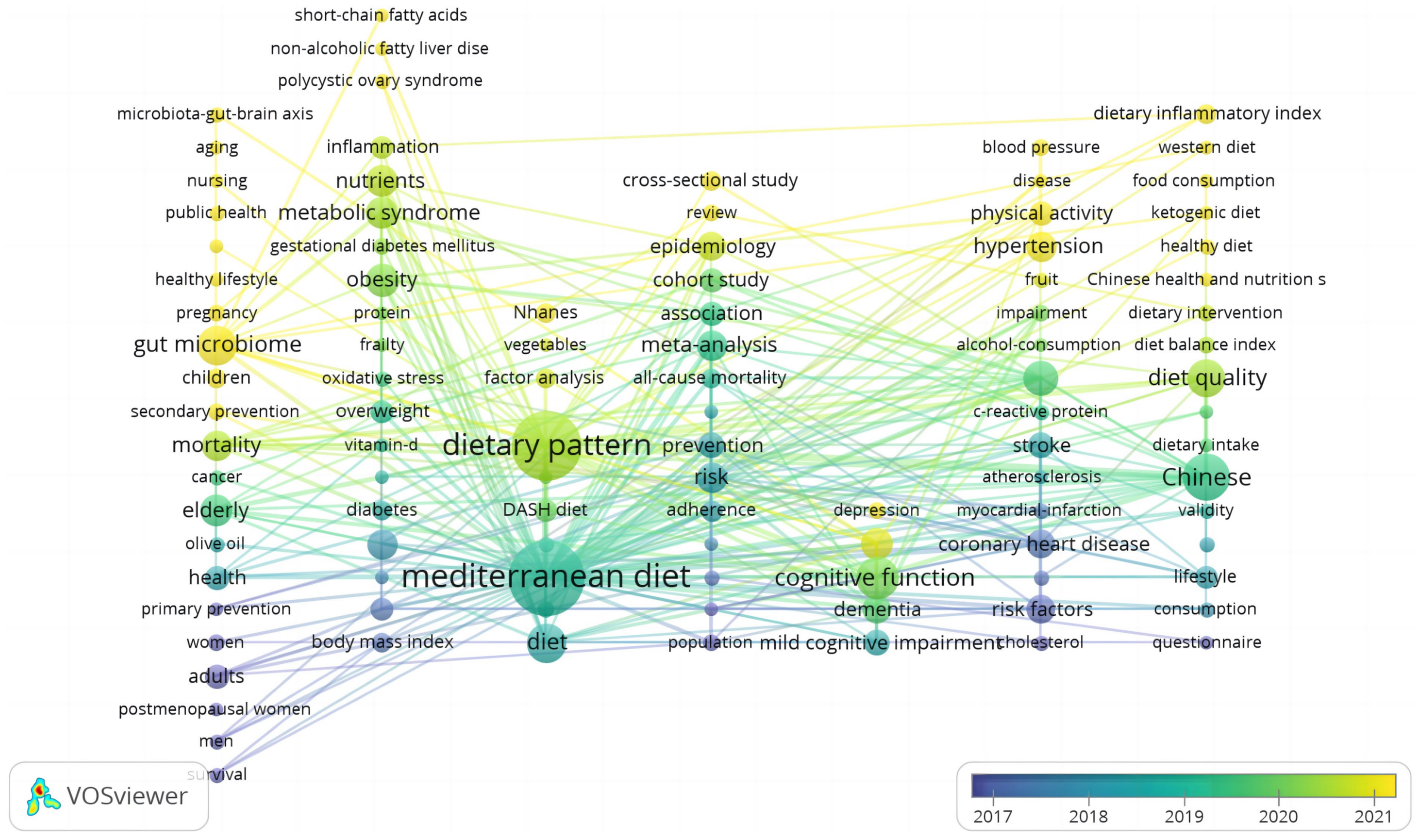
Keyword overlay map of Mediterranean-diet research in China (2006–2025). Node size, keyword frequency; node color, average publication year (blue, earlier, yellow, recent).

To trace thematic evolution, we integrated results from LDA (K = 7, C_V = 0.53) and a VOSviewer-generated keyword overlay map (Figure 5). The literature was segmented into three distinct phases based on dominant topics and term frequency distributions (Table 3).

**Table 3:** Phase-wise evolution of Mediterranean-diet research in China (2006–2025).

Phase	Years	Dominant research focus	Representative keywords / topics	Corpus share (%)
I—Pioneering	2006–2013	Cardiovascular replication of landmark Mediterranean trials	cholesterol; LDL; blood-pressure; PREVEND; PREDIMED	17
II—Public-health expansion	2014–2019	Obesity & diabetes burden; early Jiangnan-diet adaptation	BMI; diabetes risk; diet quality; rice-oil ratio; Jiangnan diet	35
III—Cross-disciplinary innovation	2020–2025	Microbiome, digital adherence, sustainability	gut microbiota; smartphone-app coaching; life-cycle assessment; carbon footprint	48

Corpus share % = proportion of total papers assigned to the dominant LDA topic(s) within each phase.

Phase I (2006–2013) is characterized by replication of Western cardiometabolic trials, with key terms such as “cholesterol” and “blood pressure.” Phase II (2014–2019) marks a pivot toward obesity and diabetes in the general population, accompanied by early mentions of the Jiangnan Diet. Phase III (2020–2025) introduces new thematic domains—gut microbiota, smartphone-based coaching, and life-cycle assessment (LCA)—reflecting broader trends in microbiomics, digital health, and sustainability. Notably, the share of publications focused on sustainability rose from under 2% in 2014 to 11% in 2024. This evolution signals a paradigm shift from biomedical replication toward culturally and technologically integrated systems-level research.

### Bibliographic coupling analysis: emergence of five citation schools

**Figure 6:**
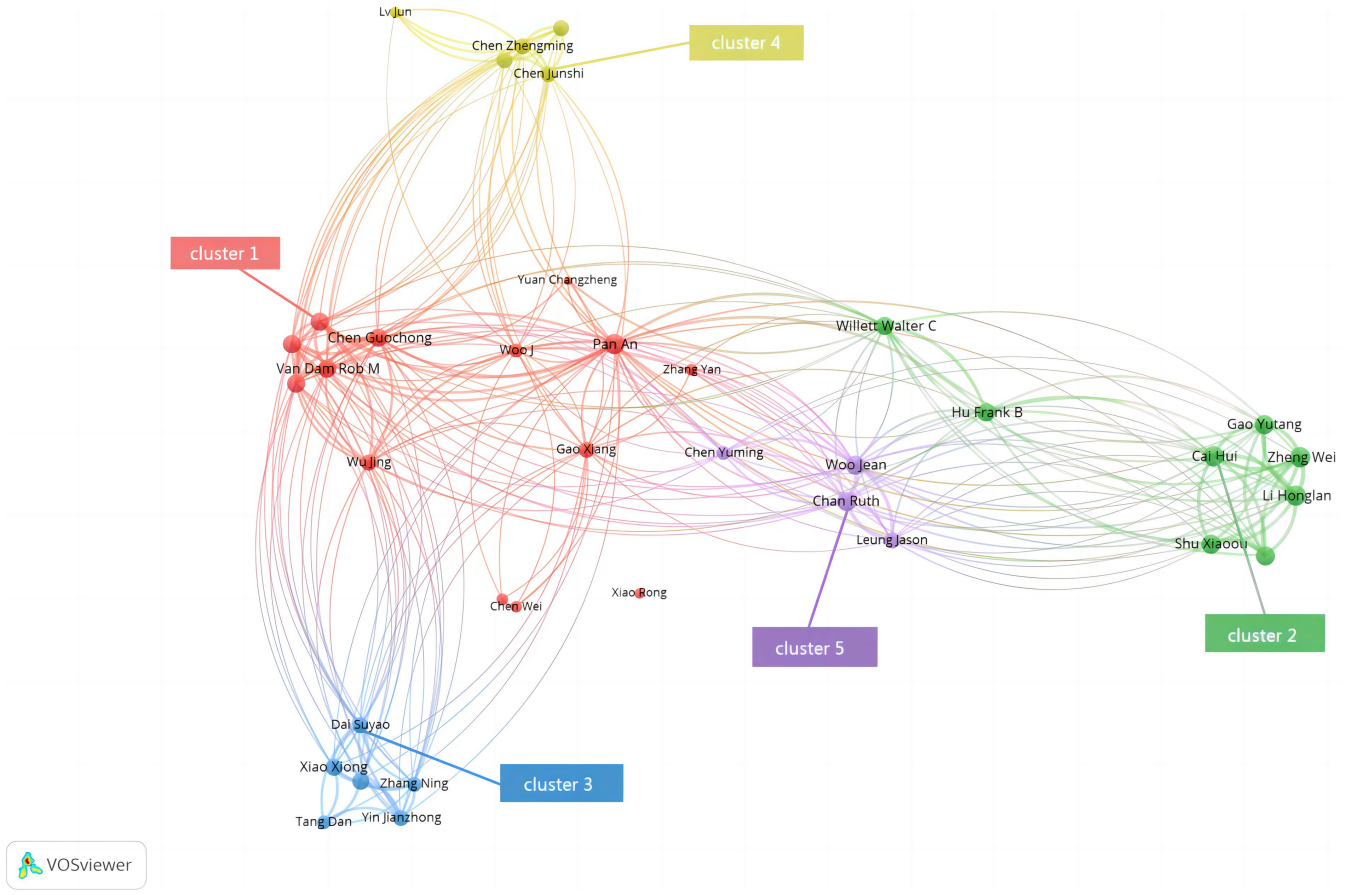
Bibliographic-coupling network of Mediterranean-diet research in China (2006–2025). Nodes represent individual papers that share≥20 references; node size, local citation count; edge width, number of shared references; colors denote five citation schools.

Bibliographic coupling (threshold: ≥20 shared references) revealed five major citation clusters (Figure 6). Cluster 1 (red) forms the central intellectual axis, anchored by Pan A., Chen Guochong, and van Dam R.M., and now includes Jiangnan Diet papers by Wang J., indicating convergence between localized dietary research and classical cardiometabolic replication.

Cluster 2 (green), led by Hu F.B. and China Kadoorie Biobank collaborators, includes large-scale epidemiological studies validating MedDiet scoring systems. Cluster 3 (blue) aggregates pediatric and community-intervention research by Xiao Xiong and Tang Dan. Cluster 4 (yellow) includes policy-oriented studies by Chen Junshi and Lv Jun that connect scientific literature to national nutrition guidelines. Cluster 5 (purple) focuses on cognitive and neuro-nutritional research but lacks Jiangnan-related content.

Network modularity (Louvain score = 0.41) indicates moderately distinct yet interconnected knowledge domains. Cross-cluster citation strength increased from 0.18 in 2006–2013 to 0.33 in 2020–2025, reflecting increasing interdisciplinarity and convergence of research aims.

### Semantic drift captured by term co-occurrence

**Figure 7:**
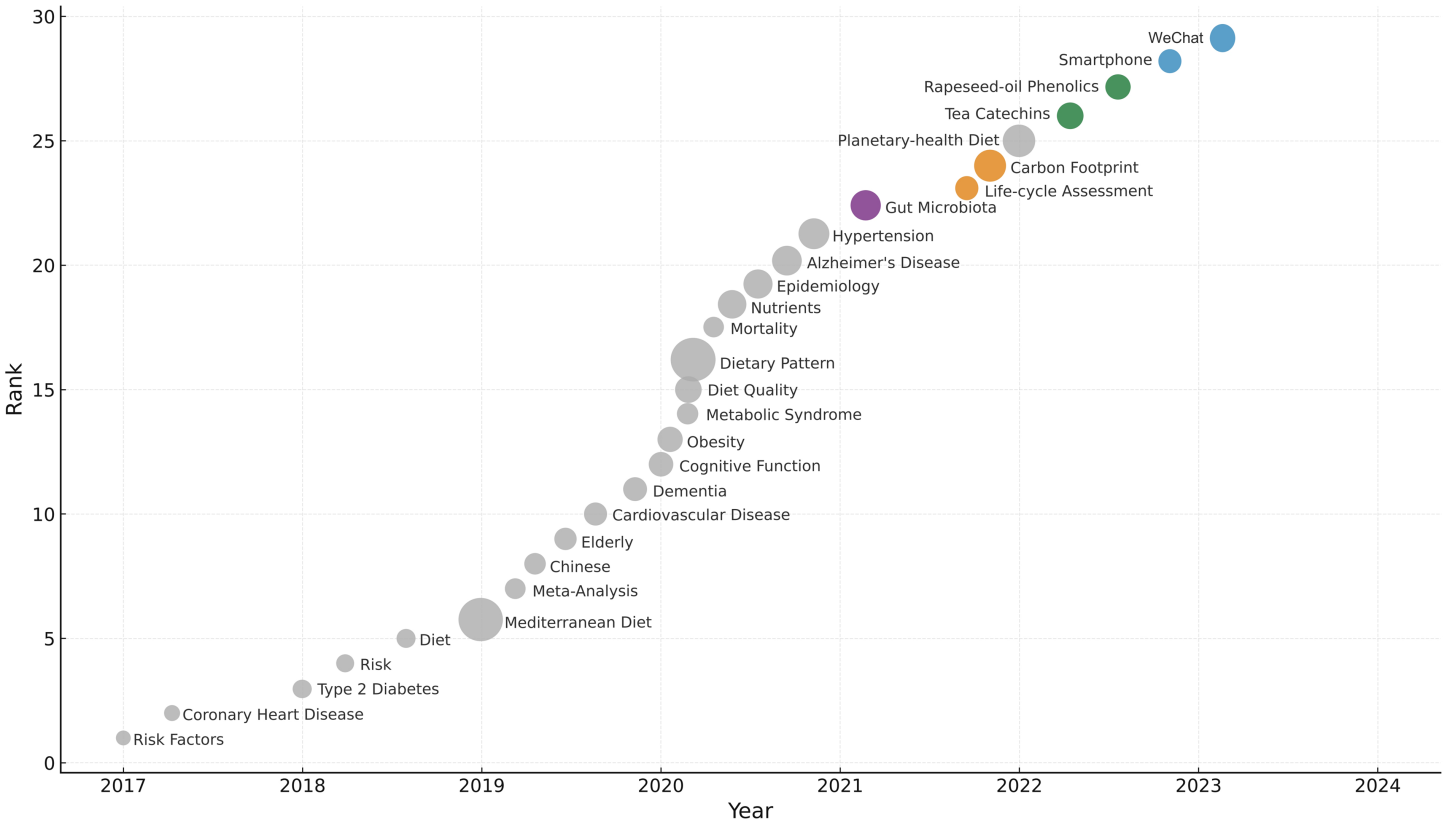
Bubble-rank timeline of emerging keywords in Mediterranean-diet research in China (2017–2024).

To explore finer-grained shifts in vocabulary, term co-occurrence analysis (TCA) was performed across four time slices (2006–2010, 2011–2015, 2016–2020, 2021–2025). Key trends emerged in Figure 7.

Ingredient localization is evident in the lexical replacement of “olive oil + phenolics” with “rapeseed-oil phenolics” and “tea catechins,” indicating substitution of Mediterranean staples with local alternatives. Digital health terminology emerges post-2020, with frequent co-occurrences of “smartphone + adherence,” “m-health,” and “precision nutrition,” reflecting integration of mobile technologies into dietary research.

Sustainability discourse gains traction through the emergence of term-pairs such as “carbon footprint + life-cycle assessment” and “planetary-health diet + greenhouse gas,” which appear predominantly after 2020. Finally, microbiome research has evolved beyond general terms like “dysbiosis” to more mechanistic language, including “short-chain fatty acids” and “bile-acid metabolism.”

Collectively, TCA illustrates the conceptual maturation of the field, with vocabulary shifting from replication-oriented jargon to sustainability and localization-focused terminology.

## Discussion

This study provides the first comprehensive bibliometric and semantic network analysis of Chinese Mediterranean diet (MedDiet) research published between 2006 and 2025. Three major trends emerge. First, the field has undergone sustained and accelerating expansion, with a CAGR of 23%, surpassing global benchmarks for MedDiet literature. Second, the thematic scope has shifted from classical cardiometabolic replication to a diversified portfolio including gut microbiome science, digital-health adherence, and sustainability. Third, bibliographic-coupling analysis reveals convergence among previously siloed citation communities, with culturally adapted Jiangnan-diet studies now integrated into the intellectual core of the field.

### Integrated synthesis of findings

Chinese MedDiet scholarship demonstrates a structured and multidimensional maturation trajectory. Quantitatively, publication output increased dramatically from a single article in 2006 to 76 in 2022, with FWCI (1.34) indicating above-average global influence. The sharp increase in Mediterranean diet publications after 2020 likely reflects heightened attention to dietary quality and immune resilience during the COVID-19 pandemic, when evidence-based dietary models attracted growing interest among clinicians and researchers. The subsequent decline after 2022 may be explained by the normalization of research priorities in the post-pandemic period, a shift in funding toward urgent domestic nutrition challenges (e.g., obesity, diabetes, food security), and increased scholarly engagement with alternative paradigms such as plant-based diets and the Jiangnan dietary model. These dynamics underscore that Mediterranean diet scholarship in China is shaped by evolving public health agendas and broader scientific trends, rather than following a simple linear trajectory.

Geographically, research capacity remains coastal-centric, led by Beijing, Hong Kong, and the Zhejiang–Shanghai corridor, yet cross-provincial collaboration has risen from 31 to 44%, reflecting broader dissemination of expertise.

Qualitatively, the field is no longer defined solely by cardiometabolic endpoints. Seven keyword clusters highlight the emergence of new domains such as microbiome–diet interactions, mobile-app–based adherence strategies, and environmental co-benefits of dietary reform. Temporal analyses confirm a three-phase evolution: initial cardiometabolic replication (2006–2013), expansion to public-health priorities and localization (2014–2019), and cross-disciplinary innovation (2020–2025). Bibliographic-coupling data reveal that Jiangnan-diet papers have transitioned from the periphery to the central clinical-citation school, signaling epistemic convergence.

Lexical drift analysis further corroborates this transformation: “olive oil + phenolics” pairs give way to “rapeseed-oil phenolics” and “tea catechins”; “smartphone + adherence” enters the lexicon; and “life-cycle assessment” and “carbon footprint” gain prominence after 2020. These findings collectively point to the emergence of a distinctive, China-adapted MedDiet paradigm that is digitally enabled, ecologically aligned, and culturally embedded.

### Biological rationale of the Mediterranean diet

Beyond publication trajectories, the health benefits of the Mediterranean diet are grounded in a synergistic matrix of nutrients and bioactive compounds. Polyphenols and carotenoids from fruits, vegetables, and wine exert potent antioxidant and anti-inflammatory effects (23); omega-3 fatty acids from fish and nuts reduce cardiometabolic risk and support cognitive function (24); monounsaturated fats from olive oil improve lipid metabolism (25); and dietary fiber from whole grains and legumes enhances glycemic control and fosters microbiome diversity (26). Collectively, these mechanisms underpin the Mediterranean diet’s cardiometabolic and neuroprotective effects and help explain the intensifying research attention in China (8). Their alignment with pressing national health challenges—such as obesity, diabetes, and healthy aging—further underscores the translational relevance of this dietary model.

### Interpretation in the context of previous work

Prior global bibliometric surveys reported CAGR estimates of 11–14% for MedDiet research up to 2020 (27, 28). The higher growth rate observed in China (23%) is largely attributable to post-2020 surges in microbiome and sustainability topics. Our analysis extends and deepens findings by Wang et al. (13) who documented initial trials of rapeseed-oil substitution but lacked network-level evidence of its mainstream adoption. Internationally, microbiome-oriented MedDiet studies began rising in 2018 (29), while China’s trajectory intensified after 2020, coinciding with the Precision Medicine Initiative and national investments in omics infrastructure (30).

### Mechanistic and contextual drivers

Three macro-level forces appear to be shaping the field’s trajectory. First, policy alignment: the “Healthy China 2030” initiative endorses salt–oil ratio reform and plant-forward dietary models, explicitly referencing the Mediterranean diet as a template for chronic disease prevention (31). Second, digital infrastructure: with over 1.1 billion WeChat users, China offers a uniquely scalable platform for mobile health (mHealth) interventions (32). The rise of “smartphone + adherence” term-pairs in our corpus reflects the academic uptake of such tools (33). Third, ecological imperatives: the national dual-carbon pledge (peak carbon by 2030, neutrality by 2060) has stimulated interest in life-cycle metrics and carbon-footprint accounting in the food system (34). This aligns with recent findings that nearly half of food-system emissions originate during primary production (35), reinforcing the relevance of dietary shifts to climate goals.

### Practical and policy implications

#### Clinical practice

Evidence suggests that phenolic-rich rapeseed oil offers anti-inflammatory benefits comparable to extra-virgin olive oil, while tea catechins may provide antioxidant capacities similar to those found in traditional Mediterranean herbs and wine (36, 37). These findings make it clinically viable and culturally acceptable to prescribe a “Chinese-adapted” Mediterranean diet that substitutes regionally familiar ingredients without compromising therapeutic bioactivity. Such adaptations could lower both cost and adherence barriers in routine cardiometabolic care.

#### Public health and policy

Randomized controlled trials have demonstrated that WeChat-delivered nutrition coaching can enhance dietary and medication adherence among patients with chronic conditions (38), Provincial Centers for Disease Control (CDCs) could embed these interventions within existing mHealth infrastructure to reach large populations at minimal incremental cost. At the national policy level, our results support incorporating a “Jiangnan-style Mediterranean pattern” into future editions of the Chinese Dietary Guidelines (13). Moreover, standardizing rapeseed oil as the reference edible fat in life-cycle assessment frameworks would harmonize public health nutrition with China’s dual-carbon agenda (39), enabling synergistic gains in health and climate policy.

### Strengths and limitations

This study adheres to the PRISMA-2020 extension for scoping reviews and bibliometric analyses, providing a transparent workflow and open-access data pipeline (40). The dual-language strategy (WoS and CNKI) enhances comprehensiveness relative to English-only reviews (41). Methodologically, triangulation across three analytical lenses—keyword co-occurrence, bibliographic coupling, and term co-occurrence—allowed for robust cross-validation of thematic, intellectual, and lexical patterns.

Limitations include: (i) the exclusion of 4% of articles indexed only in local journals outside Scopus, which may marginally underestimate citation metrics; (ii) temporal lag in citation-based metrics, which may underrepresent the coupling strength of recent publications (2024–2025); (iii) removal of very recent papers in the bibliographic coupling analysis due to short reference lists; and (iv) restriction of semantic analyses to titles and abstracts, which may miss full-text nuance, particularly in digital implementation studies.

### Future research agenda

First, future studies should integrate traditional epidemiological endpoints with life-cycle assessment to quantify the dual health-climate benefits of a Jiangnan-adapted Mediterranean dietary pattern. Nested cohort designs could assess correlations between diet-related carbon footprints and clinical outcomes, as recently piloted in large-scale European studies (42).

Second, mobile-health research should extend beyond short-term pilots. Cluster-randomized trials of ≥24 months are needed to evaluate whether app-based coaching can sustain behavioral change and reduce major cardiovascular events. Adaptive interventions incorporating gamification, push notifications, and peer support would offer insights for optimizing China’s billion-user mHealth ecosystem (38).

Finally, a multi-omics Jiangnan cohort—incorporating metagenomics, untargeted metabolomics, and food-omics (e.g., phenolic signatures of regional oils)—should be established to elucidate mechanistic pathways linking dietary polyphenols with inflammation, bile-acid metabolism, and gut–brain signaling. Recent Chinese studies involving > 900 participants demonstrate feasibility (43). Such a cohort could accelerate biomarker discovery, support personalized nutrition, and align with national digital health and climate-neutrality strategies.

## Conclusion

Over the past two decades, Chinese scholarship has transformed the Mediterranean diet (MedDiet) from a replicated cardiometabolic model into a culturally adapted, digitally enabled, and climate-conscious research paradigm. This evolution is marked by rapid publication growth (CAGR = 23%), thematic diversification into microbiome science, mobile-health interventions, and sustainability, and the convergence of previously fragmented citation communities around a Jiangnan-style adaptation.

This emergent paradigm reimagines MedDiet principles for the Chinese context—substituting olive oil with phenolic-rich rapeseed oil, in-clinic counseling with smartphone-based coaching, and nutrient-focused endpoints with integrated health–environment frameworks. Moving forward, capitalizing on this momentum through long-term digital adherence trials, coupled life-cycle assessment–epidemiology models, and multi-omics Jiangnan cohorts could yield synergistic benefits for population health and carbon neutrality.

In sum, the Mediterranean diet in China is no longer a borrowed template; it is evolving into a locally innovated platform that integrates nutrition science, digital technology, and ecological sustainability—positioning China as a global contributor to the future of dietary health.

## Data Availability

The datasets presented in this study can be found in online repositories. The names of the repository/repositories and accession number(s) can be found in the article/Supplementary material.
