# Supplementary material for: Mediterranean diet research trajectories in China (2006–2025): a scoping review and scientometric analysis to localize global nutrition models
Source: Front Nutr. 2025 Sep 24;12:1661835. doi: 10.3389/fnut.2025.1661835 (PMC12504878; doi:10.3389/fnut.2025.1661835)
Supplement: Supplementary file 3 [file Table_2.DOCX]

Supplementary Table S2：Top-10 productive and influential authors (2006–2025)

| **Rank** | **Institution** | **Documents** | **Citations** | **Cit/Doc** |
| --- | --- | --- | --- | --- |
| 1 | Willett Walter C | 6 | 439 | 73.17 |
| 2 | Pan An | 6 | 379 | 63.17 |
| 3 | Chan Ruth | 8 | 343 | 42.88 |
| 4 | Li Liming | 5 | 214 | 42.8 |
| 5 | Woo Jean | 10 | 369 | 36.9 |
| 6 | Chen Guochong | 5 | 155 | 31 |
| 7 | Gao Xiang | 6 | 49 | 8.17 |
| 8 | Xiao Xiong | 7 | 50 | 7.14 |
| 9 | Zhao Xing | 7 | 50 | 7.14 |
| 10 | Chen Yuming | 6 | 93 | 15.5 |
